# [μ-*N*,*N*′-Bis(2-pyridylmethyl­ene)ethane-1,2-diamine]bis­{aqua­[*N*,*N*′-bis­(2-pyridyl­methyl­ene)ethane-1,2-diamine]manganese(II)} tetra­kis(perchlorate)

**DOI:** 10.1107/S1600536810003764

**Published:** 2010-02-06

**Authors:** Kwang Ha

**Affiliations:** a School of Applied Chemical Engineering, The Research Institute of Catalysis, Chonnam National University, Gwangju 500-757, Republic of Korea

## Abstract

The cation of the salt, [Mn_2_(C_14_H_14_N_4_)_3_(H_2_O)_2_](ClO_4_)_4_, lies on a center of inversion, the center lying midway along the ethyl­ene chain of the bridging *N*,*N*′-bis­(2-pyridylmethyl­ene)ethane-1,2-diamine ligand. The Mn atom is chelated by two atoms N atoms of this bridging ligand, and is also coordinated by four N atoms of another ligand. The Mn atom is seven-coordinated in a penta­gonal-bipyramidal environment. The crystal structure displays inter­molecular π–π inter­actions between adjacent pyridine rings, with a shortest centroid–centroid distance of 3.784 (3) Å. The perchlorate is linked to the dinuclear cation by O—H⋯O hydrogen bonds.

## Related literature

For the crystal structures of Mn(II), Ag(I), Cu(II) and Pd(II) complexes with related ligands, see: Baar *et al.* (2001 ▶); Bowyer *et al.* (1998 ▶); Hwang & Ha (2009 ▶); Nguyen & Jeong (2006 ▶); Schoumacker *et al.* (2003 ▶).
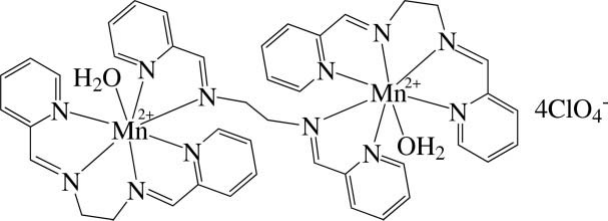


## Experimental

### 

#### Crystal data

[Mn_2_(C_14_H_14_N_4_)_3_(H_2_O)_2_](ClO_4_)_4_
*M* *_r_* = 1258.59
Monoclinic, 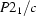

*a* = 11.3698 (6) Å
*b* = 19.026 (1) Å
*c* = 12.8628 (7) Å
β = 110.218 (1)°
*V* = 2611.1 (2) Å^3^
*Z* = 2
Mo *K*α radiation
μ = 0.77 mm^−1^
*T* = 200 K
0.24 × 0.18 × 0.12 mm

#### Data collection

Bruker SMART 1000 CCD diffractometer
Absorption correction: multi-scan (*SADABS*; Bruker, 2000 ▶) *T*_min_ = 0.873, *T*_max_ = 1.000
19343 measured reflections
6468 independent reflections
3281 reflections with *I* > 2σ(*I*)
*R*_int_ = 0.080

#### Refinement

*R*[*F* ^2^ > 2σ(*F* ^2^)] = 0.062
*wR*(*F* ^2^) = 0.178
*S* = 1.04
6468 reflections
360 parameters
2 restraints
H atoms treated by a mixture of independent and constrained refinement
Δρ_max_ = 0.82 e Å^−3^
Δρ_min_ = −0.84 e Å^−3^

### 

Data collection: *SMART* (Bruker, 2000 ▶); cell refinement: *SAINT* (Bruker, 2000 ▶); data reduction: *SAINT*; program(s) used to solve structure: *SHELXS97* (Sheldrick, 2008 ▶); program(s) used to refine structure: *SHELXL97* (Sheldrick, 2008 ▶); molecular graphics: *ORTEP-3* (Farrugia, 1997 ▶) and *PLATON* (Spek, 2009 ▶); software used to prepare material for publication: *SHELXL97*.

## Supplementary Material

Crystal structure: contains datablocks global, I. DOI: 10.1107/S1600536810003764/ng2727sup1.cif

Structure factors: contains datablocks I. DOI: 10.1107/S1600536810003764/ng2727Isup2.hkl

Additional supplementary materials:  crystallographic information; 3D view; checkCIF report

## Figures and Tables

**Table 1:** Selected bond lengths (Å)

Mn1—O1	2.215 (3)
Mn1—N6	2.257 (3)
Mn1—N3	2.278 (3)
Mn1—N2	2.278 (4)
Mn1—N5	2.280 (3)
Mn1—N1	2.504 (3)
Mn1—N4	2.639 (4)

**Table 2:** Hydrogen-bond geometry (Å, °)

*D*—H⋯*A*	*D*—H	H⋯*A*	*D*⋯*A*	*D*—H⋯*A*
O1—H1*A*⋯O3^i^	0.84 (1)	1.95 (1)	2.787 (5)	177 (6)
O1—H1*B*⋯O7^ii^	0.84 (1)	1.92 (2)	2.721 (6)	159 (6)

Symmetry codes: (i) 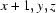; (ii) 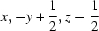.

**Fig. 1.**
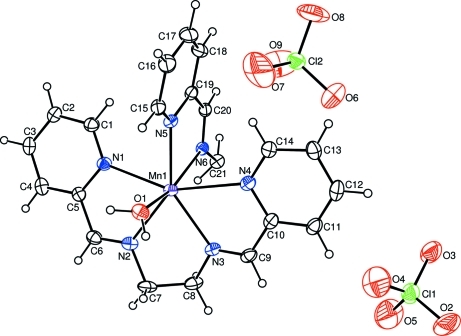
The asymmetric structure of the title compound, with displacement ellipsoids drawn at the 30% probability level for non-H atoms.

**Fig. 2.**
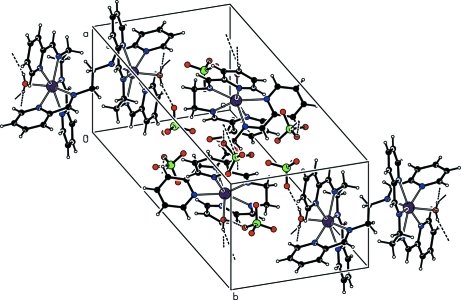
View of the unit-cell contents of the title compound. Hydrogen-bond interactions are drawn with dashed lines.

## supplementary crystallographic information

### Comment

The title compound, [Mn_2_(C_14_H_14_N_4_)_2_(H_2_O)_2_(µ-C_14_H_14_N_4_)](ClO_4_)_4_, consists of a structurally centrosymmetric dinuclear Mn^II^ complex and four perchlorate anions, and the asymmetric unit contains one half of the formula unit (Fig. 1). In the cationic complex, the two Mn^II^ ions are bridged by the symmetry-related tetradentate *N*,*N*'-bis(2-pyridylmethylene)ethane-1,2-diamine ligand, thus each Mn ion is seven-coordinated by six N atoms from the two tetradentate ligands and one O atom of the water molecule in an approximately pentagonal-bipyramidal environment, in which the five N1–N5 atoms form the pentagonal plane with O1 and N6 atoms at the apices. The six Mn—N bond lengths are considerably different and lie in the range of 2.257 (3)–2.639 (4) Å (Table 1). The N—Mn1—N chelating angles lie in the range of 66.69 (12)°–74.08 (12)° and the apical O1—Mn1—N6 bond angle is 159.64 (13)°. The crystal structure displays intermolecular π-π interactions between adjacent pyridine rings, with a shortest centroid-centroid distance of 3.784 (3) Å. The component ions interact by means of intermolecular O—H···O hydrogen bonds (Fig. 2 and Table 2).

### Experimental

To a solution of *N*,*N*'-bis(2-pyridylmethylene)ethane-1,2-diamine (0.66 g, 2.77 mmol) in EtOH (30 ml) was added Mn(ClO_4_)_2_.6H_2_O (1.00 g, 2.76 mmol) and stirred for 1 h at room temparature. The formed precipitate was separated by filtration and washed with acetone and dried under vacuum, to give a yellow powder (0.63 g). Crystals suitable for X-ray analysis were obtained by slow evaporation from a CH_3_CN solution. IR (KBr): 3394 cm^-1^ (broad).

### Refinement

H atoms were positioned geometrically and allowed to ride on their respective parent atoms [C—H = 0.95 Å (CH) or 0.99 Å (CH_2_) and U_iso_(H) = 1.2U_eq_(C)]. The H atoms of the water ligand were localized from Fourier difference maps and refined with the two restraints instructions using the following SHELXL97 (Sheldrick, 2008) command: DFIX 0.84 0.01 O1 H1A and O1 H1B.

### Crystal data

**Table:**

[Mn_2_(C_14_H_14_N_4_)_3_(H_2_O)_2_](ClO_4_)_4_	*F*(000) = 1288
*M**_r_* = 1258.59	*D*_x_ = 1.601 Mg m^−^^3^
Monoclinic, *P*2_1_/*c*	Mo *K*α radiation, λ = 0.71073 Å
Hall symbol: -P 2ybc	Cell parameters from 3474 reflections
*a* = 11.3698 (6) Å	θ = 2.2–23.2°
*b* = 19.026 (1) Å	µ = 0.77 mm^−^^1^
*c* = 12.8628 (7) Å	*T* = 200 K
β = 110.218 (1)°	Block, yellow
*V* = 2611.1 (2) Å^3^	0.24 × 0.18 × 0.12 mm
*Z* = 2	

### Data collection

**Table:**

Bruker SMART 1000 CCD diffractometer	6468 independent reflections
Radiation source: fine-focus sealed tube	3281 reflections with *I* > 2σ(*I*)
graphite	*R*_int_ = 0.080
φ and ω scans	θ_max_ = 28.3°, θ_min_ = 1.9°
Absorption correction: multi-scan (*SADABS*; Bruker, 2000)	*h* = −15→15
*T*_min_ = 0.873, *T*_max_ = 1.000	*k* = −25→22
19343 measured reflections	*l* = −12→17

### Refinement

**Table:**

Refinement on *F*^2^	Primary atom site location: structure-invariant direct methods
Least-squares matrix: full	Secondary atom site location: difference Fourier map
*R*[*F*^2^ > 2σ(*F*^2^)] = 0.062	Hydrogen site location: inferred from neighbouring sites
*wR*(*F*^2^) = 0.178	H atoms treated by a mixture of independent and constrained refinement
*S* = 1.04	*w* = 1/[σ^2^(*F*_o_^2^) + (0.0701*P*)^2^] where *P* = (*F*_o_^2^ + 2*F*_c_^2^)/3
6468 reflections	(Δ/σ)_max_ < 0.001
360 parameters	Δρ_max_ = 0.82 e Å^−^^3^
2 restraints	Δρ_min_ = −0.84 e Å^−^^3^

### Special details

**Table:**

Geometry. All esds (except the esd in the dihedral angle between two l.s. planes) are estimated using the full covariance matrix. The cell esds are taken into account individually in the estimation of esds in distances, angles and torsion angles; correlations between esds in cell parameters are only used when they are defined by crystal symmetry. An approximate (isotropic) treatment of cell esds is used for estimating esds involving l.s. planes.
Refinement. Refinement of *F*^2^ against ALL reflections. The weighted *R*-factor wR and goodness of fit *S* are based on *F*^2^, conventional *R*-factors *R* are based on *F*, with *F* set to zero for negative *F*^2^. The threshold expression of *F*^2^ > σ(*F*^2^) is used only for calculating *R*-factors(gt) etc. and is not relevant to the choice of reflections for refinement. *R*-factors based on *F*^2^ are statistically about twice as large as those based on *F*, and *R*- factors based on ALL data will be even larger.

### Fractional atomic coordinates and isotropic or equivalent isotropic displacement parameters (Å^2^)

**Table:**

	*x*	*y*	*z*	*U*_iso_*/*U*_eq_	
Mn1	0.71734 (6)	0.12570 (3)	0.16693 (5)	0.0293 (2)	
O1	0.8064 (3)	0.21764 (17)	0.2697 (3)	0.0391 (8)	
H1A	0.8820 (18)	0.224 (3)	0.277 (5)	0.07 (2)*	
H1B	0.772 (4)	0.2559 (15)	0.245 (4)	0.07 (2)*	
N1	0.9113 (3)	0.07794 (18)	0.1423 (3)	0.0329 (8)	
N2	0.7713 (3)	0.18591 (17)	0.0370 (3)	0.0356 (9)	
N3	0.5461 (3)	0.18634 (17)	0.0586 (3)	0.0339 (9)	
N4	0.5358 (3)	0.13162 (17)	0.2506 (3)	0.0354 (9)	
N5	0.7905 (3)	0.06251 (17)	0.3271 (3)	0.0292 (8)	
N6	0.6439 (3)	0.01543 (17)	0.1226 (3)	0.0286 (8)	
C1	0.9807 (4)	0.0233 (2)	0.1909 (4)	0.0377 (11)	
H1	0.9533	−0.0042	0.2399	0.045*	
C2	1.0901 (4)	0.0035 (2)	0.1753 (4)	0.0398 (11)	
H2	1.1366	−0.0360	0.2130	0.048*	
C3	1.1302 (4)	0.0422 (2)	0.1039 (4)	0.0403 (11)	
H3	1.2059	0.0306	0.0921	0.048*	
C4	1.0579 (4)	0.0986 (2)	0.0496 (4)	0.0395 (11)	
H4	1.0819	0.1256	−0.0019	0.047*	
C5	0.9507 (4)	0.1147 (2)	0.0714 (4)	0.0317 (10)	
C6	0.8697 (4)	0.1738 (2)	0.0151 (4)	0.0370 (11)	
H6	0.8909	0.2022	−0.0366	0.044*	
C7	0.6868 (4)	0.2436 (2)	−0.0174 (4)	0.0446 (12)	
H7A	0.7010	0.2579	−0.0861	0.054*	
H7B	0.7013	0.2848	0.0325	0.054*	
C8	0.5549 (5)	0.2163 (2)	−0.0439 (4)	0.0466 (13)	
H8A	0.4937	0.2550	−0.0709	0.056*	
H8B	0.5365	0.1798	−0.1022	0.056*	
C9	0.4538 (4)	0.2018 (2)	0.0877 (4)	0.0375 (11)	
H9	0.3927	0.2339	0.0442	0.045*	
C10	0.4393 (4)	0.1714 (2)	0.1861 (4)	0.0332 (10)	
C11	0.3316 (4)	0.1822 (2)	0.2109 (4)	0.0420 (12)	
H11	0.2674	0.2122	0.1658	0.050*	
C12	0.3181 (4)	0.1489 (3)	0.3016 (5)	0.0460 (13)	
H12	0.2442	0.1551	0.3193	0.055*	
C13	0.4139 (4)	0.1066 (2)	0.3659 (4)	0.0432 (12)	
H13	0.4071	0.0818	0.4277	0.052*	
C14	0.5207 (4)	0.1013 (2)	0.3373 (4)	0.0395 (11)	
H14	0.5881	0.0737	0.3837	0.047*	
C15	0.8513 (4)	0.0865 (2)	0.4293 (4)	0.0337 (10)	
H15	0.8819	0.1334	0.4378	0.040*	
C16	0.8714 (4)	0.0463 (3)	0.5229 (4)	0.0474 (13)	
H16	0.9131	0.0654	0.5944	0.057*	
C17	0.8300 (5)	−0.0219 (3)	0.5110 (4)	0.0552 (14)	
H17	0.8423	−0.0507	0.5743	0.066*	
C18	0.7702 (4)	−0.0484 (2)	0.4056 (4)	0.0438 (12)	
H18	0.7442	−0.0962	0.3955	0.053*	
C19	0.7489 (4)	−0.0046 (2)	0.3157 (4)	0.0309 (10)	
C20	0.6763 (4)	−0.0278 (2)	0.2030 (4)	0.0315 (10)	
H20	0.6530	−0.0758	0.1899	0.038*	
C21	0.5679 (4)	−0.0107 (2)	0.0132 (3)	0.0328 (10)	
H21A	0.5740	−0.0626	0.0116	0.039*	
H21B	0.5996	0.0088	−0.0435	0.039*	
Cl1	0.08812 (12)	0.30361 (7)	0.26378 (13)	0.0569 (4)	
O2	−0.0050 (5)	0.3509 (2)	0.2687 (4)	0.0965 (16)	
O3	0.0551 (3)	0.2349 (2)	0.2846 (5)	0.0951 (17)	
O4	0.2057 (4)	0.3200 (3)	0.3388 (5)	0.129 (2)	
O5	0.0906 (5)	0.3027 (3)	0.1535 (5)	0.128 (2)	
Cl2	0.59876 (11)	0.10876 (6)	0.70262 (10)	0.0395 (3)	
O6	0.4879 (4)	0.1474 (3)	0.6685 (5)	0.118 (2)	
O7	0.6953 (6)	0.1538 (3)	0.7445 (6)	0.160 (3)	
O8	0.6025 (5)	0.0622 (2)	0.7867 (4)	0.115 (2)	
O9	0.6180 (7)	0.0730 (3)	0.6164 (4)	0.153 (3)	

### Atomic displacement parameters (Å^2^)

**Table:**

	*U*^11^	*U*^22^	*U*^33^	*U*^12^	*U*^13^	*U*^23^
Mn1	0.0341 (4)	0.0249 (3)	0.0255 (4)	−0.0003 (3)	0.0060 (3)	0.0016 (3)
O1	0.046 (2)	0.0335 (19)	0.038 (2)	−0.0055 (16)	0.0145 (17)	−0.0053 (15)
N1	0.035 (2)	0.036 (2)	0.026 (2)	0.0007 (16)	0.0077 (16)	0.0027 (16)
N2	0.045 (2)	0.031 (2)	0.031 (2)	0.0033 (17)	0.0137 (18)	0.0018 (15)
N3	0.036 (2)	0.030 (2)	0.032 (2)	0.0051 (16)	0.0068 (17)	0.0027 (15)
N4	0.033 (2)	0.032 (2)	0.037 (2)	0.0020 (16)	0.0070 (17)	−0.0012 (17)
N5	0.0282 (18)	0.0297 (19)	0.027 (2)	0.0031 (15)	0.0061 (15)	0.0006 (15)
N6	0.0263 (18)	0.0294 (19)	0.028 (2)	−0.0009 (15)	0.0069 (15)	−0.0027 (15)
C1	0.039 (3)	0.040 (3)	0.034 (3)	0.002 (2)	0.012 (2)	0.004 (2)
C2	0.033 (2)	0.042 (3)	0.042 (3)	0.004 (2)	0.011 (2)	−0.001 (2)
C3	0.030 (2)	0.048 (3)	0.045 (3)	−0.006 (2)	0.015 (2)	−0.011 (2)
C4	0.037 (3)	0.049 (3)	0.035 (3)	−0.009 (2)	0.015 (2)	−0.009 (2)
C5	0.032 (2)	0.035 (2)	0.027 (2)	−0.0035 (19)	0.0090 (19)	−0.0028 (18)
C6	0.043 (3)	0.037 (3)	0.030 (3)	−0.004 (2)	0.012 (2)	0.0022 (19)
C7	0.064 (3)	0.033 (3)	0.040 (3)	0.011 (2)	0.022 (3)	0.016 (2)
C8	0.053 (3)	0.045 (3)	0.037 (3)	0.014 (2)	0.010 (2)	0.014 (2)
C9	0.034 (2)	0.026 (2)	0.044 (3)	0.0029 (19)	0.003 (2)	−0.003 (2)
C10	0.029 (2)	0.028 (2)	0.037 (3)	−0.0018 (18)	0.005 (2)	−0.0100 (19)
C11	0.034 (3)	0.032 (3)	0.056 (3)	0.003 (2)	0.011 (2)	−0.006 (2)
C12	0.031 (2)	0.048 (3)	0.060 (4)	−0.005 (2)	0.017 (2)	−0.016 (3)
C13	0.040 (3)	0.042 (3)	0.051 (3)	−0.009 (2)	0.019 (2)	−0.005 (2)
C14	0.035 (2)	0.042 (3)	0.041 (3)	−0.001 (2)	0.012 (2)	0.001 (2)
C15	0.031 (2)	0.043 (3)	0.025 (2)	−0.0028 (19)	0.0078 (19)	−0.0011 (19)
C16	0.042 (3)	0.066 (4)	0.030 (3)	−0.005 (3)	0.008 (2)	0.002 (2)
C17	0.050 (3)	0.075 (4)	0.032 (3)	−0.005 (3)	0.004 (2)	0.022 (3)
C18	0.045 (3)	0.044 (3)	0.040 (3)	−0.004 (2)	0.011 (2)	0.014 (2)
C19	0.025 (2)	0.033 (2)	0.034 (3)	0.0033 (18)	0.0093 (19)	0.0007 (18)
C20	0.028 (2)	0.025 (2)	0.042 (3)	0.0006 (17)	0.014 (2)	−0.0002 (19)
C21	0.036 (2)	0.033 (2)	0.027 (2)	−0.0004 (19)	0.008 (2)	−0.0072 (18)
Cl1	0.0494 (8)	0.0436 (8)	0.0833 (11)	0.0007 (6)	0.0301 (8)	−0.0063 (7)
O2	0.112 (4)	0.066 (3)	0.127 (4)	0.047 (3)	0.060 (3)	0.009 (3)
O3	0.051 (2)	0.049 (3)	0.179 (5)	0.0034 (19)	0.032 (3)	0.017 (3)
O4	0.069 (3)	0.104 (4)	0.175 (6)	−0.042 (3)	−0.006 (3)	−0.006 (4)
O5	0.146 (5)	0.158 (5)	0.107 (5)	0.044 (4)	0.078 (4)	0.011 (4)
Cl2	0.0421 (7)	0.0335 (6)	0.0423 (7)	−0.0021 (5)	0.0139 (5)	0.0033 (5)
O6	0.063 (3)	0.126 (4)	0.161 (6)	0.037 (3)	0.036 (3)	0.055 (4)
O7	0.127 (5)	0.116 (4)	0.208 (7)	−0.086 (4)	0.022 (5)	0.007 (4)
O8	0.174 (5)	0.092 (3)	0.122 (4)	0.061 (3)	0.106 (4)	0.072 (3)
O9	0.262 (8)	0.137 (5)	0.064 (4)	0.096 (5)	0.062 (4)	−0.001 (3)

### Geometric parameters (Å, °)

**Table:**

Mn1—O1	2.215 (3)	C8—H8A	0.9900
Mn1—N6	2.257 (3)	C8—H8B	0.9900
Mn1—N3	2.278 (3)	C9—C10	1.452 (6)
Mn1—N2	2.278 (4)	C9—H9	0.9500
Mn1—N5	2.280 (3)	C10—C11	1.382 (6)
Mn1—N1	2.504 (3)	C11—C12	1.383 (7)
Mn1—N4	2.639 (4)	C11—H11	0.9500
O1—H1A	0.840 (10)	C12—C13	1.377 (7)
O1—H1B	0.837 (10)	C12—H12	0.9500
N1—C1	1.325 (5)	C13—C14	1.390 (6)
N1—C5	1.343 (5)	C13—H13	0.9500
N2—C6	1.266 (5)	C14—H14	0.9500
N2—C7	1.467 (5)	C15—C16	1.378 (6)
N3—C9	1.264 (5)	C15—H15	0.9500
N3—C8	1.471 (6)	C16—C17	1.370 (7)
N4—C14	1.318 (6)	C16—H16	0.9500
N4—C10	1.355 (5)	C17—C18	1.384 (7)
N5—C15	1.337 (5)	C17—H17	0.9500
N5—C19	1.352 (5)	C18—C19	1.379 (6)
N6—C20	1.272 (5)	C18—H18	0.9500
N6—C21	1.461 (5)	C19—C20	1.466 (6)
C1—C2	1.379 (6)	C20—H20	0.9500
C1—H1	0.9500	C21—C21^i^	1.517 (8)
C2—C3	1.372 (6)	C21—H21A	0.9900
C2—H2	0.9500	C21—H21B	0.9900
C3—C4	1.385 (6)	Cl1—O4	1.387 (5)
C3—H3	0.9500	Cl1—O2	1.408 (4)
C4—C5	1.376 (6)	Cl1—O3	1.411 (4)
C4—H4	0.9500	Cl1—O5	1.429 (6)
C5—C6	1.476 (6)	Cl2—O7	1.349 (5)
C6—H6	0.9500	Cl2—O9	1.381 (5)
C7—C8	1.511 (6)	Cl2—O8	1.387 (4)
C7—H7A	0.9900	Cl2—O6	1.393 (4)
C7—H7B	0.9900		
			
O1—Mn1—N6	159.64 (13)	N2—C7—H7B	110.4
O1—Mn1—N3	94.76 (13)	C8—C7—H7B	110.4
N6—Mn1—N3	98.80 (12)	H7A—C7—H7B	108.6
O1—Mn1—N2	81.80 (12)	N3—C8—C7	107.5 (4)
N6—Mn1—N2	116.87 (13)	N3—C8—H8A	110.2
N3—Mn1—N2	71.73 (13)	C7—C8—H8A	110.2
O1—Mn1—N5	85.88 (12)	N3—C8—H8B	110.2
N6—Mn1—N5	74.08 (12)	C7—C8—H8B	110.2
N3—Mn1—N5	141.99 (13)	H8A—C8—H8B	108.5
N2—Mn1—N5	145.21 (13)	N3—C9—C10	121.5 (4)
O1—Mn1—N1	96.96 (12)	N3—C9—H9	119.3
N6—Mn1—N1	84.01 (11)	C10—C9—H9	119.3
N3—Mn1—N1	135.25 (13)	N4—C10—C11	122.4 (4)
N2—Mn1—N1	67.51 (12)	N4—C10—C9	116.3 (4)
N5—Mn1—N1	81.98 (12)	C11—C10—C9	121.3 (4)
O1—Mn1—N4	89.06 (12)	C10—C11—C12	119.5 (4)
N6—Mn1—N4	82.51 (11)	C10—C11—H11	120.2
N3—Mn1—N4	66.69 (12)	C12—C11—H11	120.2
N2—Mn1—N4	136.42 (12)	C13—C12—C11	118.6 (4)
N5—Mn1—N4	75.33 (11)	C13—C12—H12	120.7
N1—Mn1—N4	156.05 (11)	C11—C12—H12	120.7
Mn1—O1—H1A	114 (4)	C12—C13—C14	117.8 (5)
Mn1—O1—H1B	114 (4)	C12—C13—H13	121.1
H1A—O1—H1B	105 (5)	C14—C13—H13	121.1
C1—N1—C5	116.4 (4)	N4—C14—C13	125.0 (4)
C1—N1—Mn1	129.2 (3)	N4—C14—H14	117.5
C5—N1—Mn1	114.3 (3)	C13—C14—H14	117.5
C6—N2—C7	120.9 (4)	N5—C15—C16	122.9 (4)
C6—N2—Mn1	123.7 (3)	N5—C15—H15	118.5
C7—N2—Mn1	115.3 (3)	C16—C15—H15	118.5
C9—N3—C8	119.7 (4)	C17—C16—C15	118.8 (5)
C9—N3—Mn1	123.9 (3)	C17—C16—H16	120.6
C8—N3—Mn1	115.9 (3)	C15—C16—H16	120.6
C14—N4—C10	116.6 (4)	C16—C17—C18	119.2 (5)
C14—N4—Mn1	132.4 (3)	C16—C17—H17	120.4
C10—N4—Mn1	110.9 (3)	C18—C17—H17	120.4
C15—N5—C19	118.1 (4)	C19—C18—C17	119.1 (4)
C15—N5—Mn1	127.7 (3)	C19—C18—H18	120.5
C19—N5—Mn1	113.3 (3)	C17—C18—H18	120.5
C20—N6—C21	118.2 (4)	N5—C19—C18	121.8 (4)
C20—N6—Mn1	114.7 (3)	N5—C19—C20	116.6 (4)
C21—N6—Mn1	127.1 (3)	C18—C19—C20	121.6 (4)
N1—C1—C2	124.4 (4)	N6—C20—C19	121.0 (4)
N1—C1—H1	117.8	N6—C20—H20	119.5
C2—C1—H1	117.8	C19—C20—H20	119.5
C3—C2—C1	118.5 (4)	N6—C21—C21^i^	110.0 (4)
C3—C2—H2	120.8	N6—C21—H21A	109.7
C1—C2—H2	120.8	C21^i^—C21—H21A	109.7
C2—C3—C4	118.5 (4)	N6—C21—H21B	109.7
C2—C3—H3	120.8	C21^i^—C21—H21B	109.7
C4—C3—H3	120.8	H21A—C21—H21B	108.2
C5—C4—C3	118.9 (4)	O4—Cl1—O2	112.6 (3)
C5—C4—H4	120.6	O4—Cl1—O3	109.4 (3)
C3—C4—H4	120.6	O2—Cl1—O3	109.3 (3)
N1—C5—C4	123.3 (4)	O4—Cl1—O5	110.4 (4)
N1—C5—C6	115.6 (4)	O2—Cl1—O5	109.0 (3)
C4—C5—C6	121.1 (4)	O3—Cl1—O5	106.0 (3)
N2—C6—C5	118.6 (4)	O7—Cl2—O9	107.5 (5)
N2—C6—H6	120.7	O7—Cl2—O8	107.0 (4)
C5—C6—H6	120.7	O9—Cl2—O8	110.0 (3)
N2—C7—C8	106.6 (4)	O7—Cl2—O6	108.3 (4)
N2—C7—H7A	110.4	O9—Cl2—O6	112.6 (4)
C8—C7—H7A	110.4	O8—Cl2—O6	111.3 (3)
			
O1—Mn1—N1—C1	104.0 (4)	N2—Mn1—N6—C20	144.2 (3)
N6—Mn1—N1—C1	−55.5 (4)	N5—Mn1—N6—C20	−0.2 (3)
N3—Mn1—N1—C1	−152.0 (3)	N1—Mn1—N6—C20	83.1 (3)
N2—Mn1—N1—C1	−177.9 (4)	N4—Mn1—N6—C20	−77.0 (3)
N5—Mn1—N1—C1	19.2 (4)	O1—Mn1—N6—C21	171.0 (3)
N4—Mn1—N1—C1	0.5 (5)	N3—Mn1—N6—C21	39.8 (3)
O1—Mn1—N1—C5	−74.2 (3)	N2—Mn1—N6—C21	−34.1 (3)
N6—Mn1—N1—C5	126.3 (3)	N5—Mn1—N6—C21	−178.5 (3)
N3—Mn1—N1—C5	29.8 (4)	N1—Mn1—N6—C21	−95.2 (3)
N2—Mn1—N1—C5	3.9 (3)	N4—Mn1—N6—C21	104.7 (3)
N5—Mn1—N1—C5	−159.0 (3)	C5—N1—C1—C2	1.6 (6)
N4—Mn1—N1—C5	−177.7 (3)	Mn1—N1—C1—C2	−176.6 (3)
O1—Mn1—N2—C6	96.9 (4)	N1—C1—C2—C3	−0.6 (7)
N6—Mn1—N2—C6	−74.5 (4)	C1—C2—C3—C4	−1.1 (7)
N3—Mn1—N2—C6	−165.3 (4)	C2—C3—C4—C5	1.7 (6)
N5—Mn1—N2—C6	26.4 (5)	C1—N1—C5—C4	−0.9 (6)
N1—Mn1—N2—C6	−4.2 (3)	Mn1—N1—C5—C4	177.6 (3)
N4—Mn1—N2—C6	176.8 (3)	C1—N1—C5—C6	177.9 (4)
O1—Mn1—N2—C7	−80.0 (3)	Mn1—N1—C5—C6	−3.7 (5)
N6—Mn1—N2—C7	108.6 (3)	C3—C4—C5—N1	−0.8 (7)
N3—Mn1—N2—C7	17.9 (3)	C3—C4—C5—C6	−179.4 (4)
N5—Mn1—N2—C7	−150.4 (3)	C7—N2—C6—C5	−179.4 (4)
N1—Mn1—N2—C7	179.0 (3)	Mn1—N2—C6—C5	3.9 (6)
N4—Mn1—N2—C7	−0.1 (4)	N1—C5—C6—N2	0.3 (6)
O1—Mn1—N3—C9	−79.5 (3)	C4—C5—C6—N2	179.1 (4)
N6—Mn1—N3—C9	85.3 (3)	C6—N2—C7—C8	139.5 (4)
N2—Mn1—N3—C9	−159.2 (4)	Mn1—N2—C7—C8	−43.6 (4)
N5—Mn1—N3—C9	10.0 (4)	C9—N3—C8—C7	133.0 (4)
N1—Mn1—N3—C9	175.7 (3)	Mn1—N3—C8—C7	−39.0 (4)
N4—Mn1—N3—C9	7.5 (3)	N2—C7—C8—N3	51.7 (5)
O1—Mn1—N3—C8	92.2 (3)	C8—N3—C9—C10	177.8 (4)
N6—Mn1—N3—C8	−103.0 (3)	Mn1—N3—C9—C10	−10.9 (6)
N2—Mn1—N3—C8	12.5 (3)	C14—N4—C10—C11	2.1 (6)
N5—Mn1—N3—C8	−178.4 (3)	Mn1—N4—C10—C11	179.1 (3)
N1—Mn1—N3—C8	−12.7 (4)	C14—N4—C10—C9	−177.0 (4)
N4—Mn1—N3—C8	179.1 (3)	Mn1—N4—C10—C9	0.1 (4)
O1—Mn1—N4—C14	−91.4 (4)	N3—C9—C10—N4	6.4 (6)
N6—Mn1—N4—C14	70.0 (4)	N3—C9—C10—C11	−172.6 (4)
N3—Mn1—N4—C14	173.0 (4)	N4—C10—C11—C12	−3.0 (7)
N2—Mn1—N4—C14	−168.5 (4)	C9—C10—C11—C12	176.0 (4)
N5—Mn1—N4—C14	−5.4 (4)	C10—C11—C12—C13	0.9 (7)
N1—Mn1—N4—C14	13.7 (6)	C11—C12—C13—C14	1.8 (7)
O1—Mn1—N4—C10	92.2 (3)	C10—N4—C14—C13	0.8 (7)
N6—Mn1—N4—C10	−106.4 (3)	Mn1—N4—C14—C13	−175.4 (3)
N3—Mn1—N4—C10	−3.4 (3)	C12—C13—C14—N4	−2.8 (7)
N2—Mn1—N4—C10	15.2 (3)	C19—N5—C15—C16	−1.0 (6)
N5—Mn1—N4—C10	178.2 (3)	Mn1—N5—C15—C16	167.8 (3)
N1—Mn1—N4—C10	−162.6 (3)	N5—C15—C16—C17	1.6 (7)
O1—Mn1—N5—C15	3.9 (3)	C15—C16—C17—C18	0.4 (7)
N6—Mn1—N5—C15	−172.5 (4)	C16—C17—C18—C19	−2.8 (7)
N3—Mn1—N5—C15	−88.6 (4)	C15—N5—C19—C18	−1.6 (6)
N2—Mn1—N5—C15	73.1 (4)	Mn1—N5—C19—C18	−171.9 (3)
N1—Mn1—N5—C15	101.5 (3)	C15—N5—C19—C20	176.5 (4)
N4—Mn1—N5—C15	−86.2 (3)	Mn1—N5—C19—C20	6.1 (4)
O1—Mn1—N5—C19	173.0 (3)	C17—C18—C19—N5	3.5 (7)
N6—Mn1—N5—C19	−3.3 (3)	C17—C18—C19—C20	−174.5 (4)
N3—Mn1—N5—C19	80.6 (3)	C21—N6—C20—C19	−177.9 (4)
N2—Mn1—N5—C19	−117.7 (3)	Mn1—N6—C20—C19	3.6 (5)
N1—Mn1—N5—C19	−89.3 (3)	N5—C19—C20—N6	−6.9 (6)
N4—Mn1—N5—C19	82.9 (3)	C18—C19—C20—N6	171.2 (4)
O1—Mn1—N6—C20	−10.7 (5)	C20—N6—C21—C21^i^	102.6 (5)
N3—Mn1—N6—C20	−141.9 (3)	Mn1—N6—C21—C21^i^	−79.2 (5)

Symmetry codes: (i) −*x*+1, −*y*, −*z*.

### Hydrogen-bond geometry (Å, °)

**Table:**

*D*—H···*A*	*D*—H	H···*A*	*D*···*A*	*D*—H···*A*
O1—H1A···O3^ii^	0.84 (1)	1.95 (1)	2.787 (5)	177 (6)
O1—H1B···O7^iii^	0.84 (1)	1.92 (2)	2.721 (6)	159 (6)

Symmetry codes: (ii) *x*+1, *y*, *z*; (iii) *x*, −*y*+1/2, *z*−1/2.
